# Potential of Propolis Extract as a Natural Antioxidant and Antimicrobial in Gelatin Films Applied to Rainbow Trout (*Oncorhynchus mykiss*) Fillets

**DOI:** 10.3390/foods9111584

**Published:** 2020-11-01

**Authors:** Ilknur Ucak, Rowida Khalily, Celia Carrillo, Igor Tomasevic, Francisco J. Barba

**Affiliations:** 1 Animal Production and Technologies Department, Faculty of Agricultural Sciences and Technologies, Nigde Omer Halisdemir University, 51240 Nigde, Turkey; khalilyrowida@gmail.com; 2 Nutrition and Food Science, Faculty of Science, Universidad de Burgos, 09001 Burgos, Spain; ccarrillo@ubu.es; 3 Department of Animal Source Food Technology, Faculty of Agriculture, University of Belgrade, Nemanjina 6, 11080 Belgrade, Serbia; tbigor@agrif.bg.ac.rs; 4 Nutrition and Food Science Area, Department of Preventive Medicine and Public Health, Food Science, Toxicology and Forensic Medicine, Faculty of Pharmacy, Universitat de València, Avda Vicent Andrés Estellés, s/n. 46100 Burjassot València, Spain; francisco.barba@uv.es

**Keywords:** edible films, natural extract, antioxidants, fish quality, lipid oxidation, microbial quality

## Abstract

Usage of edible films and coatings alone or incorporated with natural extracts are a new approach to preservation and packaging of food. In this study, therefore, the microbiological, chemical quality, and sensorial changes of rainbow trout fillets coated with gelatin films supplemented with propolis extract (PE) (2, 8, 16%), as a source of polyphenols, were determined during 15 days of refrigerated storage (4 ± 1 °C). According to peroxide value (PV) and thiobarbituric acid reactive substances (TBARS) assays, lipid oxidation was delayed in the fillets coated with gelatin films incorporated with PE comparing with the control and gelatin-coated (without PE) fillets. The total volatile basic nitrogen (TVB-N) value of rainbow trout fillets showed an increase in all groups at the end of storage, observing the lowest values in the fillets coated with gelatin films prepared with 16% PE. Gelatin films enriched with PE had great inhibitory effects on the microbial growth in rainbow trout fillets. The addition of PE enhanced the effectiveness of gelatin films and delayed the lipid oxidation and sensory and microbial deterioration in trout fillets coated with these films. Thus, PE can be recommended to be used as a natural antioxidant and antimicrobial additive with gelatin films to maintain rainbow trout fillet quality.

## 1. Introduction

Rainbow trout (*Oncorhynchus mykiss*, Walbaum) is an important fish species in worldwide aquaculture, having a high nutritional value and consumed typically both fresh and frozen. The production of rainbow trout has grown exponentially over the last years reaching ≈110 thousand tonnes in Turkey mainly due to its desirable characteristics. However, the high water content, neutral pH, and high amount of polyunsaturated fatty acids make the trout susceptible to spoilage. Therefore, preservation techniques are required in order to maintain fish quality and freshness. Typically, temperature-based preservation techniques such as cooling and freezing are efficient in reducing the deterioration and extending the shelf life of fish [1,2]. However, some modifications in fish quality such as texture, color, and organoleptic properties still have been triggered during these processes [2]. Therefore, preservation techniques should be combined with active packaging and natural food additives in order to delay the decline of product quality and to achieve extended shelf life [3].

Recently, researchers have tried various biopolymer films and coatings alone/or in combination with plant additives to improve the shelf life of fish products [4]. Edible films and coatings such as chitosan, gelatin, alginate, and whey proteins show antibacterial, antifungal, and antioxidant activity, being an effective and eco-friendly way for maintaining the safety of a product during storage [5,6]. Gelatin is one of the most frequently used biodegradable polymers in the formation of films and coatings, which has barrier properties against lipids [7]. Furthermore, because of its biophysical, rheological, and functional properties, gelatin is extensively studied and highly applicable in the industry [8]. Propolis, perceived as a natural antioxidant and antibacterial source, is collected by honeybees from plant sources [9]. Propolis contains a wide variety of chemical compounds such as polyphenols, coumarins, sesquiterpene quinines, and amino acids, which can be considered as a chemical weapon against pathogens [9]. One of the most important groups are flavonoids, which represent around 50% of the propolis content. In addition to the functional properties of propolis, its use in food preservation is limited due to its resinous and bitter taste. For this reason, in this study, to avoid the direct use of propolis, a mixture of gelatin film–propolis was prepared that could exhibit reasonable sensory characteristics with extended shelf life. There are some studies related with the effects of both ethanolic and water extracts of propolis in fish fillets [10,11]. However, the incorporation of edible films with propolis extract is limited and conducted on chitosan films [12]. To the best of our knowledge, there is no study on the effects of gelatin-based edible films incorporated with propolis extract on fish fillets.

Therefore, the aim of this study was to increase the shelf life and to improve the overall quality of rainbow trout fillets wrapped with gelatin-based edible films incorporated with propolis extract. In order to assess the effectiveness of the gelatin film and incorporation of gelatin film with different concentrations of propolis extract, pH, lipid oxidation, total volatile basic nitrogen (TVB-N), microbial growth, and sensory changes were studied on trout fillets during 15 days under refrigeration (4 ± 1 °C).

## 2. Materials and Methods

### 2.1. Samples

Rainbow trout (*Oncorhynchus mykiss*) fillets were provided from a local fish farm located in Nigde (Turkey) and immediately (within 1 h) transported to the laboratory using ice boxes. Fish samples were gutted, beheaded, filleted, and washed. The fillets were divided into five groups and wrapped according to method of Ahmad et al. [13] with slight modifications. Dried films were removed from the foam dishes and sterilized under ultraviolet (UV) for 10 min. Fish fillets wrapped with gelatin (i) without propolis extract (PE) film were identified as GF, (ii) with gelatin film containing 2% PE named as P2, (iii) with gelatin film containing 8% PE named as P8, (iv) with gelatin film containing 16% PE named as P16 and one group left as control without wrapping. Each fillet was coated on both sides. Then all samples were put in the sterile foam plate, covered with stretch film (as a packaging material) and stored at 4 ± 1 °C for 15 days.

### 2.2. Propolis Extraction and Preparation of Gelatin Films

Propolis was obtained from a local market in Nigde, Turkey. It was ground into powder with a laboratory blender. Then, propolis powder and ethanol (70%) were put in a conical flash (1:10, *w*:*v*) and shaken for 5 h at 25 °C in a water bath. Then, the extracts were filtered and evaporated using a rotary evaporator at 45 °C.

The method previously described by Gomez-Estaca et al. [14] was used for the preparation of gelatin films with slight modifications. Glycerol (0.1 mL per g of gelatin) and D-sorbitol (0.15 g per g of gelatin) were added to the dissolved gelatin solution in distilled water (25 °C, for 30 min with shaking) (8 g/100 mL) and kept at 45 °C for 15 min. Propolis extracts (PE) were added to the film solution at different concentrations (2%, 8%, and 16% by volume per mass of gelatin). Film solutions were poured into square polystyrene foam dishes as 40 mL and put into a cabine for drying at room temperature for 48 h at 50% relative humidity.

### 2.3. Total Phenolic Content and Antioxidant Activity

The Folin–Ciocalteu colorimetric method was used for the determination of total phenolic content (TPC) of the propolis extracts [15]. Results were expressed as mg gallic acid equivalents/g sample (mg GAE/g sample). PE antioxidant activity was determined by means of the photometric method described by Re et al. [16]. The results were expressed in µmol Trolox equivalents (TE)/g sample.

### 2.4. Physicochemical Analysis

The pH values of the samples were determined by dipping the pH-meter probe into the fish homogenates (1:1, *w*:*v*, fish:distilled water).

For the determination of total volatile basic nitrogen (TVB-N), the method of Schormuller [17] was used. Homogenized fish samples (10 g) were put into the flask with the addition of 1 mg magnesium oxide. Then, the samples were boiled for 20 min and distilled into 10 mL of 0.1 mol equi/L HCl solution in a flask and Tashiro indicator was added. The flasks were titrated with 0.1 mol equi/L NaOH after distillation. The results were expressed as mg nitrogen/100 g sample.

The AOAC (Association of Official Analytical Chemists) method [18] was used in order to determine the peroxide value (PV). Two grams of sample were mixed with 30 mL of a solution consisting of glacial acetic acid:chloroform (2:3, *v*/*v*). After that, 1 mL of saturated potassium iodide (KI) solution was added, and the mixture was put under darkness for 5 min. The mixture was taken and 75 mL of distilled water were added; then the mixture was titrated with sodium thiosulfate (Na_2_S_2_O_3_) (0.1 M) with the addition of a starch solution as an indicator. The results were calculated as meq O_2_/kg.

A thiobarbituric acid reactive substances (TBARS) assay was determined according to the AOCS (American Oil Chemists’ Society) [19] method. Lipid dissolved in n-butanol was mixed with the same amount of TBA reagent (0.288 g/100 mL). The absorbance of the samples was measured with an UV–VIS spectrophotometer at 530 nm after incubation at 95 °C for 120 min in a water bath for the color reactions. Results were expressed as follows:

TBARS (mg malondialdehyde (MDA)/kg) = 50 × (absorbance of lipid − absorbance of blank)/sample weight (mg)

### 2.5. Microbiological Analysis

Ten grams of fish samples were mixed with 90 mL sterile ringer solution. Other decimal serial dilutions were used from this homogenate. Plate count agar (PCA) was used for the determination of total psychrophilic bacteria and total viable counts. Then, the plates were incubated at 8 °C for 7 days and 37 °C for 24–48 h, respectively. Yeast and mold were determined by plating on potato dextrose agar (PDA, pH 3.5) incubating the samples at 25 °C for 36–48 h. For the determination of Enterobacteriaceae, the pour plating method with violet red bile agar (VRBA) and incubation at 37 °C for 36–48 h was used.

### 2.6. Sensory Analysis

Sensory evaluation was conducted according to the method proposed by Amerina et al. [20] with slight modifications. Ten trained panelists aged between 25 and 35 years participated in sensory tests. The panelists were not informed about the experimental approach, and the samples were blind-coded with 3-digit random numbers. The gelatin films were removed before the fillets were served to the panelists. Separated sensory test boxes were used for the evaluation of samples under daylight at 24 °C. In order to test the samples, panelists were asked to open the plates covered with stretch film. The samples were assessed in terms of odor, texture, color, appearance, and overall acceptance on a nine-point hedonic scale during the storage period. A score of 9–7 indicated “very good”, a score of 6.9–4.0 “good”, and a score of 3.9–1.0 denoted as “spoiled”.

### 2.7. Statistical Analysis

All measurements were carried out in triplicate, and data were subjected to analysis of variance (ANOVA) and Duncan’s multiple range tests using the SPSS Version 18.0 statistical package (SPSS Inc., Chicago, IL, USA). Differences were regarded as statistically significant at *p* < 0.05. Statistical differences were marked with different letters. Capital letters indicate significant difference among groups, and lower-case letters indicate significant difference among storage days.

## 3. Results and Discussion

### 3.1. Total Phenolic Content and Antioxidant Capacity

In the present study, the value of TPC in the propolis extract was determined as 593.313 mg GAE/g, which was higher than that reported by Socha et al. [21] (150–197 mg GAE/g) and Aliyazicioglu et al. [22] (115–210 mg GAE/g). Similarly, Ozdal et al. [23] found the TPC of ethanolic extracts of Turkish propolis between 2748 and 19,970 mg GAE/100 g. In another study, Chaillou et al. [24] reported the amount of TPC in Argentina propolis between 92 and 170 mg GAE/g. Ciftci-Yilmaz [25] determined the TPC of Azerbaijan propolis extracted with ethanol (95%) between 10.94 and 79.23 mg GAE/g. Moreira et al. [26] reported that the methanolic extract of propolis samples contained 40.83–94.54 mg GAE/g total phenolic compounds.

It was stated that the phenolic compound content of propolis extract was significantly affected by the kind of solvent and concentration used [27]. Moreover, the essential factor influencing the TPC of propolis is floral origins of propolis, which exhibit various biological effects such as antioxidants and antimicrobials [28,29]. Our findings for TPC are in accordance with the results of Ahn et al. [30] and Kalogeropoulos et al. [31], who studied the TPC of Chinese propolis and Greek propolis, respectively.

The antioxidant activity of propolis extract in the present study was found to be 569.684 μmol TE/g propolis. Socha et al. [21] reported the antioxidant activity of propolis from various regions of Poland between 3.96 and 4.98 mM TE/g. Likewise Oses et al. [32] found the antioxidant capacity of Brazilian propolis (80% ethanolic extract) ranged between 25.5 and 439.2 µmol TE/g sample, while Serra-Bonvehi and Lacalle-Gutierrez [33] reported the antioxidant activity of Spanish propolis between 420 and 1430 μmol TE/g. In another study, antioxidant activity of Brazilian red propolis extracted with 70% ethanol was determined as 1223 μmol TE/g, which is higher than those of the reported studies by Oses et al. [32] and Andrea et al. [34]. Kumazawa et al. [35] and Ahn et al. [30] reported that quercetin and kaempferol, which are the most active flavonoids, contain five and four hydroxyl groups responsible for the antioxidant features. Therefore, the aforementioned factors influencing the phenolic compound content of propolis extract may also explain the wide range of antioxidant activities reported in the literature for this natural product.

### 3.2. Physicochemical Properties

First of all, the pH trend of trout fillets coated with and without gelatin films during storage at 4 °C was evaluated (Table 1). As can be seen in the table, the initial pH value of trout fillets was 6.482, which was lower than those values reported by other researchers for trout fillets [36,37,38]. It should be noted that the pH value of the control group was significantly higher (*p* < 0.05) than those fillets coated with gelatin film incorporated with PE, reaching values up to 7.548 at the end of the storage. Ludorf and Mayer [39] reported that the upper limit of pH value for fresh fish is between 6.8 and 7.0. In the present study, the pH value of trout fillets coated with gelatin film without PE reached a value of 7.524 after 15 days, while for the fillets coated with gelatin film incorporated with 8 and 16% PE, the pH values were 6.880 and 6.769, respectively. Similarly, Alparslan et al. [40] found that the initial pH value of trout samples was 6.30, which was the lowest value during the storage period in the samples coated with gelatin film enriched with laurel essential oil.

As was previously observed by other authors, the accumulation of alkaline compounds such as ammonia and trimethylamine, due to the activity of spoilage bacteria, promotes an increase in the pH values [41]. Moreover, Volpe et al. [38] reported that the generally dissociation of carbonic acid results in the increase of pH as the storage time progresses. As reported in the literature, a coating can protect the surface of the fillet from the spoiling effect of oxygen, which leads to a pH increase, probably as a result of basic amine production [42].

On the other hand, the changes of TVB-N content in the trout fillets wrapped with or without gelatin films during storage period were also evaluated (Table 1). The initial TVB-N value of trout fillets was 16.804 mg N/100 g and increased gradually during the storage time for both control and gelatin film-coated fillets. TVB-N is one of the most widely used quality indexes for fish, and the limit level of TVB-N was specified as 35 mg N/100 g by the European Commission [43] guidelines. In the present study, TVB-N values of the control and GF samples exceeded the limit value at the 6th and 9th day of the storage and reached 53.903 and 52.504 mg N/100 g at the end of the storage period, respectively. The activity of spoilage bacteria in amino acids of fish muscle causes the accumulation of volatile bases, thus promoting an increase of TVB-N [44]. TVB-N values of the fillets coated with gelatin film incorporated with PE were significantly (*p* < 0.05) lower at the end of the storage. Moreover, P8 and P16 groups showed the lowest (*p* < 0.05) TVB-N values, which did not exceed the limit value until the 15th day of storage.

Nowzari et al. [45] reported that the initial TVB-N value of trout fillets was 18.78 mg N/100 g, observing significantly lower values in coated fillets during storage. Similarly, Ojagh et al. [46] found the TVB-N value of trout fillets was 12.13 mg N/100 g at the beginning of storage, while after the 16th day of the storage, the samples coated with chitosan prepared with cinnamon oil reached lower TVB-N values (14.23 mg N/100 g) compared to the control (42.93 mg N/100 g) and chitosan coated samples (22.86 mg N/100 g). Other studies indicated that the incorporation of essential oils or plant extracts with edible films and coatings inhibited the TVB-N value increases in fish fillets [4,13,40,47]. Therefore, the results obtained in the present study suggest that the incorporation of PE with gelatin film is effective to enhance film properties, most probably due to the bioactive phenolic compounds found in propolis extract.

Moreover, taking into account lipid oxidation is the main cause of fish spoilage after microbial growth [48], the peroxide value (PV), which is an indicator of the primary oxidation products such as peroxides and hydroperoxides [49], was also determined in this work. The effect of gelatin film enriched with or without PE on the changes of PV of trout fillets is shown in Table 1. At the beginning of the storage, the PV of trout fillets was 2.004 meq O_2_/kg sample. The PV of all samples increased with the elapse of storage time; however, the highest (*p* < 0.05) values were found in the control and GF groups during the storage. At the end of storage, the PVs of control, GF, P2, P8, and P16 groups were 8.503, 7.002, 6.956, 5.505, and 5.001 meq O_2_/kg, respectively. Fillets coated with gelatin films incorporated with PE showed lower PV than those of control and GF groups during the whole storage period, which means that the addition of PE is an efficient way to increase the protective effect of gelatin film to retard the production of PV in trout fillets stored at 4 °C. According to Yuan et al. [50], the application of edible films and coatings with the incorporation of antioxidants represents a new approach to solve the oxidation problem in food products. For instance, the results of the present study are in agreement with those previously reported by Rezaei and Shahbazi [51] and Alparslan et al. [40], who reported that the enrichment of chitosan and gelatin films with essential oils and plant extracts was an effective tool to control the delay in lipid oxidation.

In order to evaluate the secondary lipid oxidation degree, the thiobarbituric acid reactive substances (TBARS) assay was conducted [52]. Changes in the TBARS values of all samples are presented in Table 1. As can be seen in the table, the initial TBARS value of trout fillets was 0.882 mg MDA/kg. TBARS values of the controls and gelatin coated fillets increased with the elapse of storage time. However, the fillets coated with gelatin films enriched with PE reached significantly (*p* < 0.05) lower TBARS values in comparison with the controls or gelatin coated samples. At the 15th day of storage, TBARS values of control, GF, P2, P8, and P16 were found as 1.588, 1.558, 1.488, 1.417, and 1.284 mg MDA/kg, respectively. The maximum TBARS level of frozen or chilled fish indicating good quality is 5 mg MDA/kg of tissue [53]. In the present study, TBARS values for all groups were much lower than the maximum limits throughout the storage period. Sun et al. [47] explained the reason for this phenomena as curing of the films on the surfaces of the gelatin films and forming a protective film that made the gelatin films free from direct interaction with the oxygen, which promotes lipid oxidation.

In a previous study, Jeon et al. [52] reported lower TBARS content in chitosan-coated herring and cod fillets than those of the uncoated samples during the storage period. Moreover, Ahmad et al. [13] found a lipid oxidation delay in sea bass coated with gelatin films enriched with lemongrass essential oil (LEO). They claimed that this was the result of the antioxidant property of LEO. Similar results were found by Alparslan et al. [40], who reported lower TBARS values in trout samples coated with gelatin films incorporated with laurel essential oil than those of control and gelatin film-coated samples. Reis et al. [54] also found that the supplementation of microencapsulated propolis extract resulted in a strong suppressing effect on oxidation in burger meat. Similarly, Spinelli et al. [55] observed lower lipid oxidation in fish burger supplemented with 5% spray-dried propolis than the control samples, mainly due to the higher polyphenol content of propolis.

In this study, gelatin coated samples combined with PE showed a lower lipid oxidation in comparison with gelatin-coated samples or control samples. The addition of PE into the gelatin film probably had a synergistic effect.

### 3.3. Changes in Microbiological Quality

**Figure 1:**
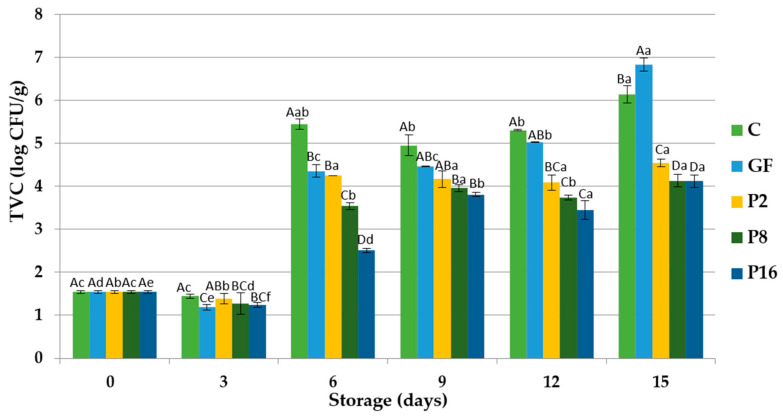
Changes in total viable counts (TVC) of rainbow trout fillets coated with gelatin films incorporated with propolis extract (PE) during storage at 4 °C. C: Control without gelatin film, GF: fillets coated with gelatin film, P2: fillets coated with gelatin film incorporated with 2% PE, P8: fillets coated with gelatin film incorporated with 8% PE, P16: fillets coated with gelatin film incorporated with 16% PE. Different capital letters indicate a significant difference among groups, and different lower-case letters indicate a significant difference among storage days (*p* < 0.05).

**Figure 2:**
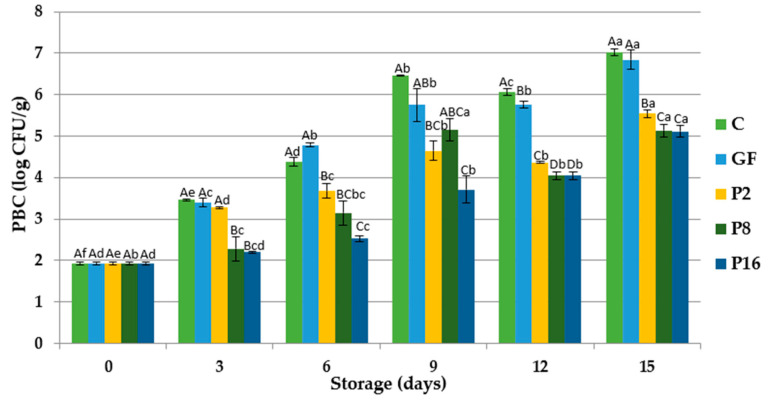
Changes in total psychrophilic bacteria counts (PBC) of rainbow trout fillets coated with gelatin films incorporated with propolis extract (PE) during storage at 4 °C. C: Control without gelatin film, GF: fillets coated with gelatin film, P2: fillets coated with gelatin film incorporated with 2% PE, P8: fillets coated with gelatin film incorporated with 8% PE, P16: fillets coated with gelatin film incorporated with 16% PE. Different capital letters indicate a significant difference among groups, and different lower-case letters indicate a significant difference among storage days (*p* < 0.05).

**Figure 3:**
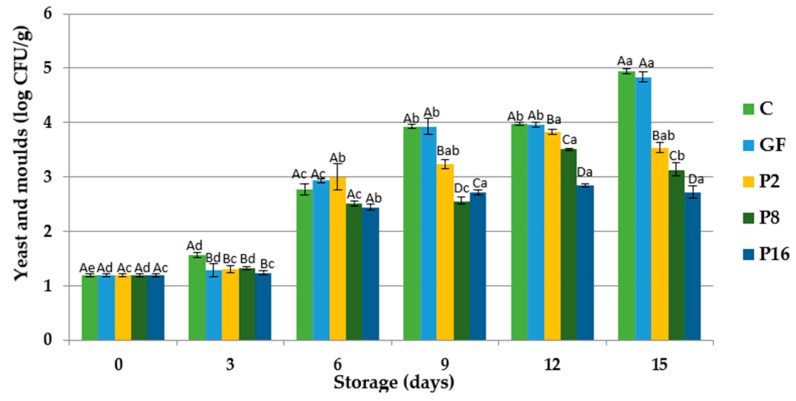
Changes in total yeast and molds of rainbow trout fillets coated with gelatin films incorporated with propolis extract (PE) during storage at 4 °C. C: Control without gelatin film, GF: fillets coated with gelatin film, P2: fillets coated with gelatin film incorporated with 2% PE, P8: fillets coated with gelatin film incorporated with 8% PE, P16: fillets coated with gelatin film incorporated with 16% PE. Different capital letters indicate a significant difference among groups, and different lower-case letters indicate a significant difference among storage days (*p* < 0.05).

**Figure 4:**
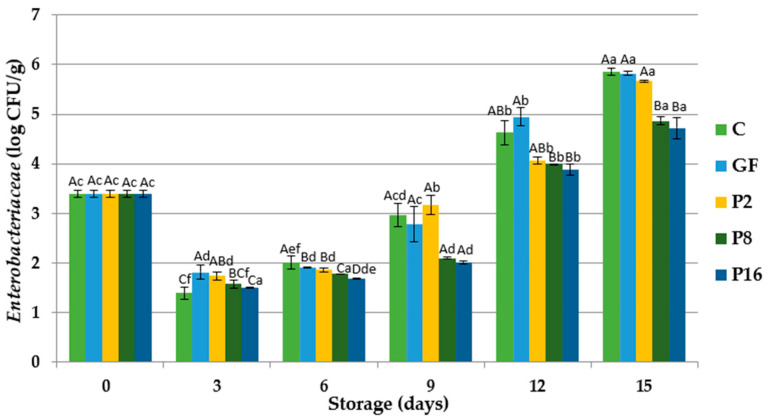
Changes in total Enterobacteriaceae counts of rainbow trout fillets coated with gelatin films incorporated with propolis extract (PE) during storage at 4 °C. C: Control without gelatin film, GF: fillets coated with gelatin film, P2: fillets coated with gelatin film incorporated with 2% PE, P8: fillets coated with gelatin film incorporated with 8% PE, P16: fillets coated with gelatin film incorporated with 16% PE. Different capital letters indicate a significant difference among groups, and different lower-case letters indicate a significant difference among storage days (*p* < 0.05).

The overall microbiological results of trout fillets wrapped with and without gelatin films are presented in Figure 1, Figure 2, Figure 3 and Figure 4. The initial total viable counts (TVCs) of trout fillet were found to be 1.543 log CFU/g (Figure 1), which were lower than values reported by other researches in trout fillets [16,28]. The TVC values of trout fillets were significantly (*p* < 0.05) different between control and gelatin coated samples during the storage period. However, lower TVCs were observed in the fillets wrapped with gelatin film supplemented with PE. For instance, significant (*p* < 0.05) lower TVCs were found in the samples coated with gelatin film incorporated with 16% PE followed by 8% PE compared with the control, GF, and P2 samples. At the end of the storage, TVC values of samples were reported as 6.14, 6.842, 4.536, 4.121, and 4.112 log CFU/g in the control, GF, P2, P8, and P16, respectively. The lower viable counts of coated fillets can be attributed to the antibacterial activity of propolis, which resulted in lower microbiological growth [56]. The antioxidant and antimicrobial properties of propolis have been largely ascribed to the polyphenolic fraction such as phenolic acids and flavonoids [57,58].

In the aerobically stored fresh fish, Gram-negative psychrotrophic bacteria are the main groups causing the spoilage [50]. The effects of gelatin coating enriched with PE was significant (*p* < 0.05) for psychrotrophic bacteria (Figure 2). At the beginning of the storage, the psychrotrophic bacteria count (PBC) of rainbow trout was 1.932 log CFU/g, showing an increase with the storage period until the 15th day. During the storage period, PBC of fillets coated with gelatin films incorporated with PE were lower than those of the gelatin film coated samples and control samples. At the 15th day of storage, PBC of trout fillets reached 7.024 log CFU/g for control samples, which is higher than the upper acceptability limit (7 log CFU/g) for fresh fish [59], while these values were determined as 6.840, 5.541, 5.120, and 5.114 log CFU/g in GF, P2, P8, and P16 groups, respectively.

In a previous study, Jouki et al. [60] monitored the initial number of PBC as 3.10 log CFU/g of rainbow trout fillets wrapped with chitosan films supplemented with oregano or thyme essential oil. The initial PBC of trout fillets coated with gelatin films incorporated with garlic peel extract (GPE) was found as 2.59 log CFU/g, which is higher than the value found in the present study, and it was reported that the treatment of gelatin films enriched with GPE had significant effects on the growth of PBC [48]. In this study, it was observed that gelatin films incorporated with PE were efficient to inhibit the psychrotrophic bacteria growth in trout fillets.

Total yeast and mold counts of rainbow trout fillets coated with gelatin films during storage at 4 °C is shown in Figure 3. The initial number of total yeast and molds was 1.194 log CFU/g, showing an increase during the storage period in all groups. At the end of the storage, total yeast and mold count of trout fillets reached 4.940, 4.841, 3.535, 3.122, and 2.273 log CFU/g in control, GF, P2, P8, and P16 groups, respectively. Results showed that the incorporation of PE with GF had a significant effect on the growth of yeast and mold, since lower values were observed in those groups (mainly in P16). In a study Petruzzi et al. [61] reported that propolis delayed the fungal and bacterial growth. Similarly, Kahramanoglu et al. [62] found that propolis is very effective in controlling pathogenic decay based on studies on its antimicrobial and fungicidal effects.

Total Enterobacteriaceae count of rainbow trout fillets are shown in Figure 4. Enterobacteriaceae is considered as a hygiene indicator and spoilage microorganisms in fresh rainbow trout. The initial number of Enterobacteriaceae was 3.390 log CFU/g. At the 3rd day of storage, the Enterobacteriaceae count of trout fillets showed a decrease in all groups, while after day 9 of storage, total Enterobacteriaceae number increased until the end of storage. At the end of storage, Enterobacteriaceae counts were 5.860, 5.822, 5.671, 4.865, and 4.724 log CFU/g in control, GF, P2, P8, and P16 groups, respectively. The lowest bacteria counts were obtained from P8 and P16 groups. This result is in agreement with other previous studies evaluating the shelf life of rainbow trout fillets coated with gelatin films enriched with laurel essential oil [40] and tilapia fillets coated with fungal chitosan containing pomegranate peel extract [49].

### 3.4. Sensory Evaluation

The sensory results of the rainbow trout fillets treated with gelatin films incorporated with PE are presented in Table 2. As expected, the sensory scores of fillets wrapped with gelatin films enriched with PE showed higher values than those of the control and GF groups. According to the sensory evaluation, control and GF samples were rejected at day 9, while P2 group sample were rejected at day 12. Moreover, P8 and P16 groups were rejected at the 15th day of the storage. The quality of trout fillets in P8 and P16 groups maintained 6 days more compared with the control and GF samples. As compared to the control and GF groups, it was found that the incorporation of PE with gelatin films showed a positive effect on the trout fillets, since P8 and P16 groups were more preferred and had higher sensory scores.

When trout fillets were unacceptable by sensory panel, the PBC values were 6.448 and 5.753 log CFU/g for the control and GF groups, respectively, at the 9th day; 4.362 log CFU/g for P2 at the 12th day; and 5.124 and 5.110 log CFU/g at the 15th day for P8 and P16 groups, respectively (Section 3.3). Therefore, the product is rejected when the microbiological quality is still acceptable, which means that the shelf life established based on sensory evaluation is shorter than that obtained from a microbiological approach.

Data reported by Jouki et al. [60] suggested a shelf life of rainbow trout fillets coated with edible film containing oregano or thyme essential oil as 18 days, while the shelf-life of the control group was 10 days. Similar results were found by Jasour et al. [63], who reported a shelf life extension after the addition of chitosan in rainbow trout fillets as 4 days compared with the control samples. Ucak [48] reported the shelf life of rainbow trout fillets as 5, 7, and 10 days in control group, gelatin film wrapped group, and gelatin film enriched with garlic peel extract wrapped group, respectively. These results related to the application of gelatin films incorporated with plant extracts or essential oils to fish fillet showed that the incorporation of these extracts improved the film properties, thus maintaining the sensory quality and therefore extending the shelf life of trout fillets.

## 4. Conclusions

After evaluating the impact of the incorporation of propolis extract (PE) with gelatin films for preservation of rainbow trout fillets, it can be concluded that the addition of PE in the gelatin films enhanced the antioxidant and antimicrobial properties of films since the lowest microbiological and chemical scores were obtained from these groups. According to sensory assessment, the shelf life of trout fillets was 6 days for the control and GF groups, 9 days for the P2 group, and 12 days for P8 and P16 groups, respectively. It can be concluded that the quality of rainbow trout fillets can be better preserved after using 8 and 16% PE in gelatin films.

## Figures and Tables

**Table 1:** Changes in physicochemical properties of rainbow trout fillets coated with gelatin films incorporated with propolis extract (PE) during storage at 4 °C.

	Storage Period (Days)	C	GF	P2	P8	P16
**pH**	**0**	6.482 ± 0.054 ^Ac^	6.482 ± 0.054 ^Ac^	6.482 ± 0.054 ^Ae^	6.482 ± 0.054 ^Abc^	6.482 ± 0.054 ^Ac^
	**3**	6.713 ± 0.041 ^Ab^	6.679 ± 0.052 ^Ab^	6.644 ± 0.022 ^ABc^	6.548 ± 0.047 ^BCc^	6.458 ± 0.079 ^Cbc^
	**6**	6.733 ± 0.040 ^Ab^	6.703 ± 0.000 ^Ab^	6.680 ± 0.027 ^Ac^	6.577 ± 0.078 ^Bbc^	6.554 ± 0.011 ^Bb^
	**9**	6.730 ± 0.102 ^Ab^	6.615 ± 0.068 ^Ab^	6.571 ± 0.024 ^Bd^	6.582 ± 0.013 ^Bb^	6.579 ± 0.014 ^Bb^
	**12**	7.504 ± 0.014 ^Aa^	7.493 ± 0.092 ^Aa^	7.309 ± 0.021 ^Bb^	6.836 ± 0.067 ^Ca^	6.713 ± 0.000 ^Da^
	**15**	7.548 ± 0.019 ^Aa^	7.524 ± 0.036 ^Aa^	7.527 ± 0.017 ^Aa^	6.880 ± 0.000 ^Ba^	6.769 ± 0.147 ^Ca^
**TVB-N** (mg N/100 g)	**0**	16.804 ± 0.000 ^Ae^	16.804 ± 0.000 ^Ae^	16.804 ± 0.000 ^Ad^	16.804 ± 0.000 ^Ae^	16.804 ± 0.000 ^Ae^
	**3**	28.003 ± 0.000 ^Ad^	21.700 ± 0.991 ^Ad^	20.303 ± 8.911 ^Ac^	18.904 ± 0.992 ^Ad^	19.647 ± 0.944 ^Ad^
	**6**	35.702 ± 0.967 ^Ac^	32.203 ± 3.955 ^ABc^	28.001 ± 0.004 ^Bb^	27.302 ± 2.974 ^Bc^	25.204 ± 1.982 ^Bcd^
	**9**	40.601 ± 0.000 ^Ab^	39.200 ± 1.983 ^Ab^	34.302 ± 0.989 ^Bcb^	32.904 ± 0.985 ^Bb^	30.802 ± 1.977 ^Bcd^
	**12**	43.400 ± 1.978 ^Ab^	44.801 ± 0.000 ^Ab^	35.704 ± 0.985 ^Bb^	34.300 ± 0.987 ^BCb^	32.203 ± 0.000 ^Cb^
	**15**	53.903 ± 2.966 ^Aa^	52.504 ± 4.954 ^Aa^	49.700 ± 2.967 ^ABa^	42.001 ± 1.983 ^BCa^	44.100 ± 0.988 ^Ca^
**PV** (meq O_2_/kg)	**0**	2.004 ± 0.002 ^Ad^	2.004 ± 0.002 ^Ac^	2.004 ± 0.002 ^Ad^	2.004 ± 0.002 ^Ac^	2.004 ± 0.002 ^Ac^
	**3**	5.002 ± 0.000 ^Ac^	5.504 ± 0.707 ^Ab^	2.004 ± 0.001 ^Bd^	1.994 ± 0.001 ^Bc^	1.974 ± 0.065 ^Bc^
	**6**	6.504 ± 0.714 ^Ab^	5.002 ± 0.002 ^Bb^	3.502 ± 0.714 ^Bc^	2.503 ± 0.714 ^Cc^	1.943 ± 0.074 ^Cc^
	**9**	5.002 ± 0.002 ^Bc^	6.502 ± 0.710 ^Aa^	5.466 ± 0.704 ^Ab^	3.978 ± 0.001 ^Cb^	3.967 ± 0.000 ^Cb^
	**12**	7.001 ± 0.000 ^Ab^	6.978 ± 0.033 ^Aa^	6.003 ± 0.002 ^Aab^	5.477 ± 0.720 ^BCa^	4.502 ± 0.712 ^Cab^
	**15**	8.503 ± 0.713 ^Aa^	7.002 ± 0.001 ^Ba^	6.956 ± 0.007 ^Bab^	5.505 ± 0.708 ^Ca^	5.001 ± 0.000 ^Ca^
**TBARS** (mg MDA/kg)	**0**	0.882 ± 0.0438 ^Ad^	0.882 ± 0.038 ^Ad^	0.882 ± 0.038 ^Ac^	0.882 ± 0.038 ^Ad^	0.882 ± 0.038 ^Ad^
	**3**	1.248 ± 0.010 ^Ac^	0.862 ± 0.112 ^Bd^	0.824 ± 0.022 ^Bc^	0.790 ± 0.040 ^Be^	0.777 ± 0.023 ^Be^
	**6**	1.217 ± 0.092 ^Ac^	1.114 ± 0.030 ^Ac^	0.912 ± 0.082 ^Bc^	1.277 ± 0.000 ^Cf^	0.704 ± 0.052 ^Cf^
	**9**	1.474 ± 0.024 ^Ab^	1.730 ± 0.000 ^Aa^	1.023 ± 0.024 ^Bb^	0.975 ± 0.000 ^Cc^	0.912 ± 0.064 ^Cc^
	**12**	1.523 ± 0.017 ^Aab^	1.493 ± 0.001 ^Bb^	1.460 ± 0.014 ^Ba^	1.252 ± 0.001 ^Cb^	1.088 ± 0.012 ^Db^
	**15**	1.588 ± 0.016 ^Aa^	1.558 ± 0.033 ^Ab^	1.488 ± 0.000 ^Ba^	1.417 ± 0.024 ^Ca^	1.284 ± 0.015 ^Da^

Means indicated by different capital letters in the same row differ significantly (*p* < 0.05). Means indicated by different lowercase letters in the same column differ significantly (*p* < 0.05). C: control without gelatin film, GF: fillets coated with gelatin film, P2: fillets coated with gelatin film incorporated with 2% PE, P8: fillets coated with gelatin film incorporated with 8% PE, P16: fillets coated with gelatin film incorporated with 16% PE.

**Table 2:** Changes in sensory scores of rainbow trout fillets coated with gelatin films incorporated with propolis extract (PE) during storage at 4 °C.

	Storage Period (Days)	C	GF	P2	P8	P16
**Odor**	**0**	9.000 ± 0.000 ^Aa^	9.000 ± 0.000 ^Aa^	9.000 ± 0.000 ^Aa^	9.000 ± 0.000 ^Aa^	9.000 ± 0.000 ^Aa^
	**3**	8.002 ± 0.530 ^Bb^	9.000 ± 0.000 ^Aa^	9.000 ± 0.000 ^Aa^	8.902 ± 0.354 ^Aa^	9.000 ± 0.000 ^Aa^
	**6**	8.124 ± 0.831 ^Bb^	8.750 ± 0.461 ^Aa^	9.000 ± 0.000 ^Aa^	8.752 ± 0.458 ^Aa^	9.000 ± 0.000 ^Aa^
	**9**	3.865 ± 0.832 ^Cc^	3.372 ± 0.923 ^Cb^	5.622 ± 1.064 ^Bb^	7.869 ± 0.643 ^Ab^	7.623 ± 0.521 ^Ab^
	**12**	1.124 ± 0.347 ^Cd^	1.366 ± 0.522 ^Cc^	3.002 ± 0.533 ^Bc^	5.251 ± 1.580 ^Ac^	5.119 ± 0.825 ^Ac^
	**15**	1.002 ± 0.000 ^Bd^	1.001 ± 0.000 ^Bc^	1.248 ± 0.458 ^Bd^	2.123 ± 0.636 ^Ad^	2.247 ± 0.458 ^Ad^
**Texture**	**0**	9.000 ± 0.000 ^Aa^	9.000 ± 0.000 ^Aa^	9.000 ± 0.000 ^Aa^	9.000 ± 0.000 ^Aa^	9.000 ± 0.000 ^Aa^
	**3**	8.253 ± 0.461 ^Bb^	9.000 ± 0.000 ^Aa^	9.000 ± 0.000 ^Aa^	8.872 ± 0.354 ^Aa^	9.000 ± 0.000 ^Aa^
	**6**	6.747 ± 0.892 ^Bc^	7.754 ± 0.462 ^Ab^	9.000 ± 0.000 ^Aa^	8.620 ± 0.522 ^Aa^	9.000 ± 0.000 ^Aa^
	**9**	3.124 ± 0.988 ^Dd^	2.251 ± 0.891 ^Ec^	5.124 ± 0.350 ^Cb^	8.502 ± 0.531 ^Aa^	7.374 ± 0.520 ^Bb^
	**12**	1.369 ± 0.743 ^BCe^	1.254 ± 0.457 ^Cd^	2.367 ± 0.521 ^Bc^	5.371 ± 1.766 ^Ab^	5.117 ± 0.991 ^Ac^
	**15**	1.002 ± 0.000 ^Be^	1.003 ± 0.001 ^Bd^	1.115 ± 0.347 ^Bd^	2.502 ± 0.763 ^Ac^	2.503 ± 0.527 ^Ad^
**Color**	**0**	9.000 ± 0.000 ^Aa^	9.000 ± 0.000 ^Aa^	9.000 ± 0.000 ^Aa^	9.000 ± 0.000 ^Aa^	9.000 ± 0.000 ^Aa^
	**3**	7.872 ± 0.636 ^Bb^	8.874 ± 0.458 ^Aab^	8.871 ± 0.348 ^Aa^	8.869 ± 0.354 ^Aa^	9.000 ± 0.000 ^Aa^
	**6**	7.001 ± 0.758 ^Bc^	8.253 ± 0.888 ^Ab^	8.623 ± 0.464 ^Aa^	8.754 ± 0.521 ^Aa^	8.752 ± 0.457 ^Aa^
	**9**	2.001 ± 0.933 ^Dd^	3.372 ± 0.921 ^Cc^	5.368 ± 0.922 ^Bb^	8.002 ± 0.758 ^Aa^	7.621 ± 0.523 ^Aa^
	**12**	1.004 ± 0.002 ^Ce^	1.367 ± 0.524 ^Cd^	2.503 ± 2.002 ^Bc^	4.617 ± 2.000 ^Ab^	4.118 ± 0.612 ^Ab^
	**15**	1.002 ± 0.000 ^Be^	1.002 ± 0.000 ^Bd^	1.117 ± 0.351 ^Bd^	2.503 ± 0.531 ^Ac^	2.254 ± 0.463 ^Ac^
**Appearance**	**0**	9.000 ± 0.000 ^Aa^	9.000 ± 0.000 ^Aa^	9.000 ± 0.000 ^Aa^	9.000 ± 0.000 ^Aa^	9.000 ± 0.000 ^Ad^
	**3**	8.000 ± 0.762 ^Bb^	8.754 ± 0.461 ^Aab^	8.753 ± 0.462 ^Aa^	8.868 ± 0.345 ^Aa^	9.000 ± 0.000 ^Aa^
	**6**	7.124 ± 0.643 ^Bc^	8.252 ± 0.887 ^Ab^	8.754 ± 0.461 ^Aa^	8.621 ± 0.743 ^Aab^	8.747 ± 0.464 ^Aa^
	**9**	2.868 ± 0.638 ^Cd^	3.121 ± 0.644 ^Cc^	5.747 ± 0.710 ^Ba^	7.872 ± 0.827 ^Ad^	7.503 ± 0.527 ^Aa^
	**12**	1.124 ± 0.352 ^Ce^	1.124 ± 0.353 ^Ce^	3.365 ± 0.922 ^Bc^	5.370 ± 1.602 ^Ac^	4.619 ± 1.192 ^Ac^
	**15**	1.000 ± 0.000 ^Be^	1.000 ± 0.000 ^Be^	1.254 ± 0.463 ^Bd^	2.366 ± 0.521 ^Ad^	2.254 ± 0.463 ^Ad^
**Overall acceptance**	**0**	9.000 ± 0.000 ^Aa^	9.000 ± 0.000 ^Aa^	9.000 ± 0.000 ^Aa^	9.000 ± 0.000 ^Aa^	9.000 ± 0.000 ^Aa^
	**3**	8.000 ± 0.532 ^Bb^	9.000 ± 0.000 ^Aa^	9.000 ± 0.000 ^Aa^	8.874 ± 0.351 ^Aa^	9.000 ± 0.000 ^Aa^
	**6**	7.000 ± 0.760 ^Bc^	8.754 ± 0.457 ^Aa^	9.000 ± 0.000 ^Aa^	8.867 ± 0.354 ^Aa^	9.000 ± 0.000 ^Aa^
	**9**	3.000 ± 0.930 ^Dd^	3.365 ± 0.743 ^Db^	6.000 ± 0.761 ^Cb^	8.370 ± 0.744 ^Aa^	7.500 ± 0.530 ^Bb^
	**12**	1.124 ± 0.351 ^Ce^	1.369 ± 0.524 ^Cc^	3.000 ± 9.533 ^Bc^	4.866 ± 1.887 ^Ab^	4.754 ± 0.461 ^Ac^
	**15**	1.000 ± 0.000 ^Be^	1.000 ± 0.000 ^Bc^	1.252 ± 0.464 ^Bd^	2.374 ± 0.521 ^Ac^	2.503 ± 0.532 ^Ad^

Means indicated by different capital letters in the same row differ significantly (*p* < 0.05). Means indicated by different lowercase letters in the same column differ significantly (*p* < 0.05). C: control without gelatin film, GF: fillets coated with gelatin film, P2: fillets coated with gelatin film incorporated with 2% PE, P8: fillets coated with gelatin film incorporated with 8% PE, P16: fillets coated with gelatin film incorporated with 16% PE.
